# Deletion of Nhlh2 Results in a Defective Torpor Response and Reduced Beta Adrenergic Receptor Expression in Adipose Tissue

**DOI:** 10.1371/journal.pone.0012324

**Published:** 2010-08-23

**Authors:** Umesh D. Wankhade, Kristen R. Vella, Dana L. Fox, Deborah J. Good

**Affiliations:** 1 Department of Human Nutrition, Foods and Exercise, Virginia Polytechnic Institute and State University, Blacksburg, Virginia, United States of America; 2 Department of Veterinary and Animal Sciences and Molecular and Cellular Biology Graduate Program, University of Massachusetts, Amherst, Massachusetts, United States of America; University of Parma, Italy

## Abstract

**Background:**

Mice with a targeted deletion of the basic helix-loop-helix transcription factor, Nescient Helix-Loop-Helix 2 (Nhlh2), display adult-onset obesity with significant increases in their fat depots, abnormal responses to cold exposure, and reduced spontaneous physical activity levels. These phenotypes, accompanied by the hypothalamic expression of Nhlh2, make the Nhlh2 knockout (N2KO) mouse a useful model to study the role of central nervous system (CNS) control on peripheral tissue such as adipose tissue.

**Methodology:**

Differences in body temperature and serum analysis of leptin were performed in fasted and *ad lib* fed wild-type (WT) and N2KO mice. Histological analysis of white (WAT) and brown adipose tissue (BAT) was performed. Gene and protein level expression of inflammatory and metabolic markers were compared between the two genotypes.

**Principal Findings:**

We report significant differences in serum leptin levels and body temperature in N2KO mice compared with WT mice exposed to a 24-hour fast, suggestive of a defect in both white (WAT) and brown adipose tissue (BAT) function. As compared to WT mice, N2KO mice showed increased serum IL-6 protein and WAT IL-6 mRNA levels. This was accompanied by slight elevations of mRNA for several macrophage markers, including expression of macrophage specific protein F4/80 in adipose, suggestive of macrophage infiltration of WAT in the mutant animals. The mRNAs for β3-adrenergic receptors (β3-AR), β2-AR and uncoupling proteins were significantly reduced in WAT and BAT from N2KO mice compared with WT mice.

**Conclusions:**

These studies implicate Nhlh2 in the central control of WAT and BAT function, with lack of Nhlh2 leading to adipose inflammation and altered gene expression, impaired leptin response to fasting, all suggestive of a deficient torpor response in mutant animals.

## Introduction

The adipokine leptin is secreted by adipose tissue in proportion to fat mass to signal fat storage reserves in the body, and mediate long-term appetite controls. Since the discovery of leptin in 1994 [1], the perspective toward adipose tissue as simply a fat storage site has changed due to the recent developments in the basic understanding of adipose tissue functions. Leptin is now seen as an adipokine that signals starvation rather than energy surplus. Leptin levels drop in response to a fast, signaling the organism to eat more and decrease energy expenditure as fat storages are low [2]. This is a protective mechanism developed by the body to mobilize energy stores for important physiological processes and reduce energy consuming processes that are not essential for immediate survival.

White adipose tissue (WAT) plays a major role in peripheral fatty acid synthesis and sequestration of triglycerides from normal circulation. Until recently, it was thought that the only role of brown adipose tissue (BAT) was in thermoregulation in newborns and hibernating animals. However recent studies using Positron Emission Tomography scans have shown active BAT depots in the cervical, supraclavicular, paravertebral, mediastinal, para-aortic and suprarenal regions of adult humans, suggesting that BAT contributes to energy balance in human adults as well [3], [4], [5].

Studies to date support a role for the sympathetic nervous system (SNS) as the primary initiator of adipose tissue functions in rodents [6], [7] and humans [8]. For example, SNS ganglia from the thoracic and lumbar regions have been shown to innervate fat pads [9]. Increased norepinephrine turnover during conditions such as cold exposure and fasting, also indicate SNS innervation of WAT [10], [11]. Cold exposure and fasting also affect adipose tissue function through decreases in leptin synthesis and release [12], [13], [14]. Even though injection of pseudorabies virus into inguinal WAT pads labels cells within the paraventricular nucleus of the hypothalamus, bilateral destruction of the paraventricular nucleus does not prevent WAT lipid mobilization following fasting [15]. As in the majority of tissues, the SNS exerts its effects on adipose tissue through the central melanocortin system. The melanocortin system may mobilize different lipid depots in the body [8], [16], [17], [18]. An injection of melanotan II, the central melanocortin receptor (Mcr) agonist, provokes a differential sympathetic drive of WAT and BAT reflected by an increased norepinephrine turnover [19]. Recently, Nogueiras and colleagues showed that chemical blockage of Mc4r results in alterations in WAT metabolism and insulin sensitivity [20]. Adipose tissue is innervated by Mc4r-positive SNS neurons, which are ultimately responsible for regulating lipid mobilization [18], [21]. Expression of both the β-adrenergic receptor (β-ARs) and uncoupling protein (UCP) family members in adipose tissue are controlled by SNS inputs [22], [23]. Although it is clear that the melanocortin signaling pathway plays an instrumental role in transmitting SNS signals to adipose and other tissues, the central or peripheral nature of this mediation is still unclear.

In our laboratory, we are studying nescient helix-loop-helix 2 (Nhlh2), a basic-helix-loop helix transcription factor expressed in the paraventricular and arcuate nuclei of the hypothalamus [24]. Expression of Nhlh2 can also be found in cranial nerves (Good, unpublished) as well as the gray matter of the cervical and thoracic areas of the spinal cord [25]. We have previously shown that Nhlh2 is a key component in the central melanocortin signaling pathway via transcriptional regulation of the prohormone convertase 1/3 (PC1/3) gene, especially following leptin stimulation [26]. Reduced PC1/3 levels in mice containing a targeted deletion of Nhlh2 (N2KO mice) leads to lower levels of α-melanocyte-stimulating-hormone (α-MSH), the melanocortin receptor-binding neuropeptide [24]. Considering the adult onset obesity in the mutant mice [27], the reduced melanocortinergic tone in N2KO mice [24], [26], and the expression patterns of Nhlh2 in the hypothalamus and spinal cord, we hypothesized that a targeted deletion of Nhlh2 would result in a disruption of the Mcr to adipose tissue outflow, leading to defects in adipose tissue metabolism. The response of N2KO mice to a fasting challenge with a particular focus on WAT and BAT function was assessed in this study. The possible contribution of inflammation in WAT to the physiological changes detected in N2KO mice was explored using markers of macrophages infiltration and inflammation. Finally, the requirement for functional, centrally-expressed Nhlh2 in expression of β-ARs and UCPs in WAT and BAT was investigated.

## Results

### Fasting Leptin Levels

**Figure 1:**
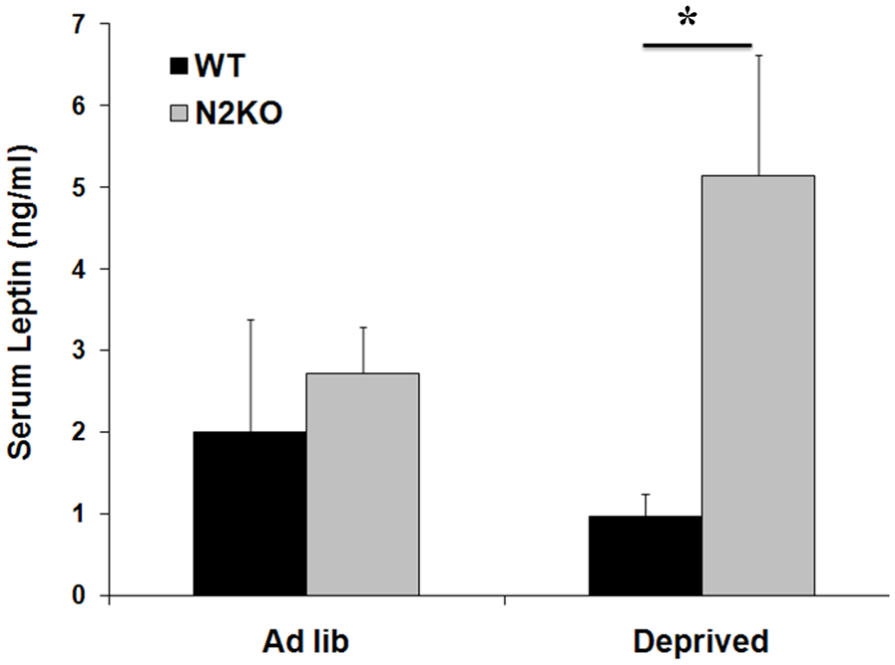
Serum leptin levels in *ad lib* fed and fasted WT and N2KO mice. Serum leptin was measured in WT and N2KO mice following *ad libitum* feeding (*Ad lib*), and following a 24 hour fast (Deprived). Data are reported as leptin concentration (pg/ml) ± SEM. (*p≤0.05).

*Ad libitum* (*ad lib*) fed N2KO mice have normal food intake and leptin levels until they become overtly obese after 20 weeks of age [27]. Furthermore, food intake does not differ significantly in *ad lib* fed WT and N2KO mice measured over two hours, or in mice given two hours of feeding following a 24-hour fast [28]. As previously reported in pre-obese mice [29] and confirmed with these experiments, *ad lib* fed N2KO mice did not show significant differences in serum leptin levels when compared to *ad lib* fed WT mice. Interestingly, fasted N2KO mice presented a significant, 5.16-fold (*p≤0.05, T value = −2.99, df = 4) increase in serum leptin levels compared to WT mice. Consistent with expected results, fasted WT mice showed reduced serum leptin levels compared to *ad lib* fed WT mice (Fig 1).

### Core Body Temperature Following Fasting

**Figure 2:**
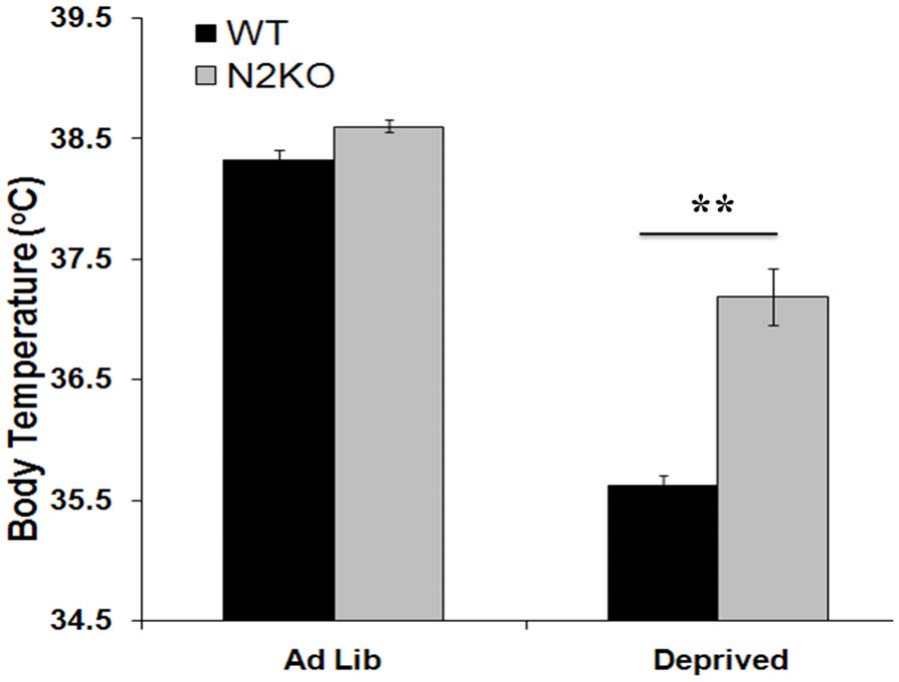
Core body temperature in *ad lib* fed and fasted WT and N2KO mice. Body temperature (°C) was measured using a rectal thermometer in WT and N2KO mice following *ad libitum* feeding (*Ab lib*) and following a 24-hour fast (Deprived). (**p≤0.01).

Normal mice subjected to fasting, or an energy deficit, will decrease their body temperature to conserve energy. This behavior is known as torpor [30]. A failure to reduce leptin can impair the ability of mice to enter torpor [31]. Since N2KO mice did not reduce serum leptin levels with fasting, body temperature was analyzed as an indicator of torpor. N2KO mice have the same core body temperature as WT mice under *ad lib* feeding conditions (Fig 2), consistent with previous results [32]. WT mice dropped their body temperature approximately 3°C during the 24 hour fast as expected (Fig 2) [33]. N2KO mice only showed a 1°C drop in body temperature with fasting (Fig 2). This 2°C difference between WT and N2KO mice is significant between the two genotypes (*p<0.001, T value = −6.40, df = 2).

### WAT and BAT morphology

**Figure 3:**
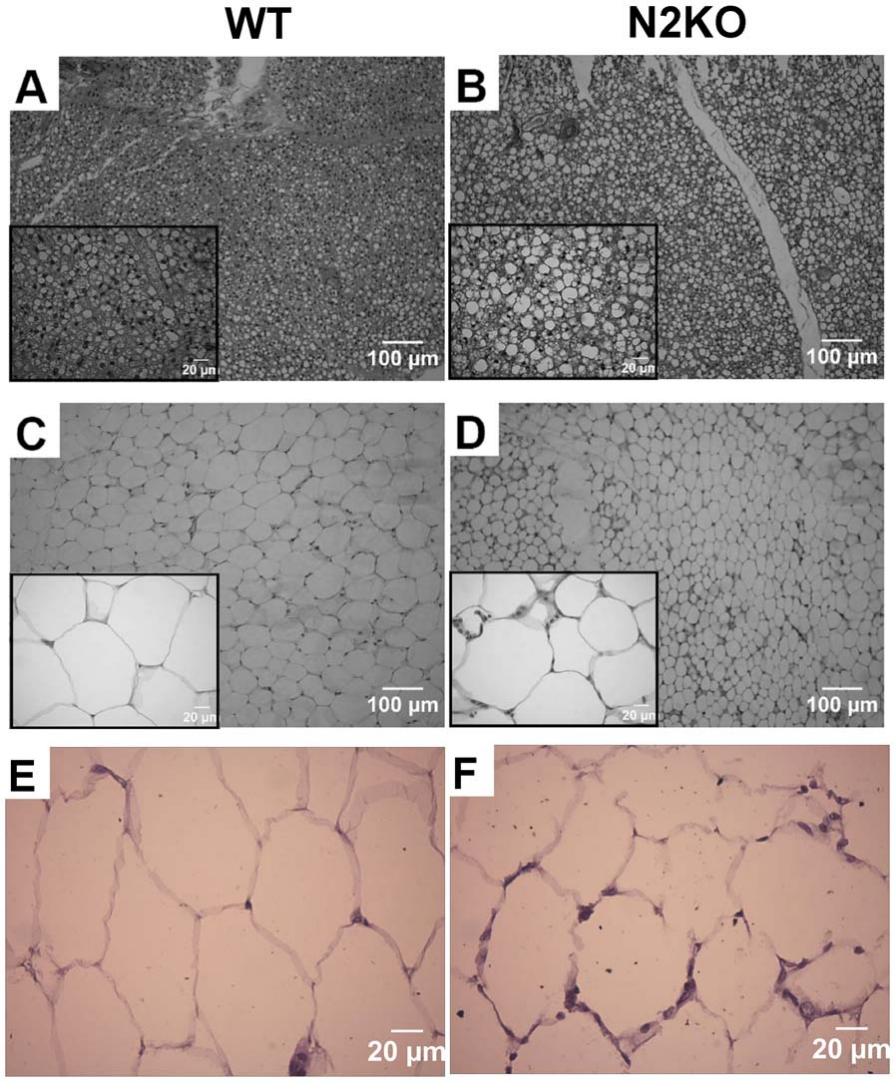
Histology and immunohistochemistry of Brown and White Adipose Tissues from N2KO and WT mice. H&E staining of Brown Adipose Tissue (**A**) or White Adipose Tissue (**C**) from WT, (**B**) and (**D**) from N2KO. Scale bars for whole and inset pictures are given. Immunohistochemistry of WAT using F4/80, a macrophage specific marker staining of WT (**E**) and N2KO (**F**).

Based on the impaired leptin response and body temperature in fasted N2KO animals, WAT and BAT morphology was examined. Hematoxylin and eosin (H&E) stained BAT from N2KO mice shows a vacuolated appearance (Fig 3A–B), while inset images shows larger lipid vacuoles in BAT From N2KO compared to the smaller vacuoles in WT. WAT from N2KO and WT mice shows an increased presence of smaller dense cells infiltrating between regions of the adipose cells in the N2KO mice (Fig 3C–D). Inset image shows individual adipocytes and adipocytes surrounded by small dense cells in N2KO mice. F4/80 staining in N2KO mice suggests that these small dense cells are macrophages (Fig 3E–F).

### Analysis of Nhlh2 expression in BAT and WAT

**Figure 4:**
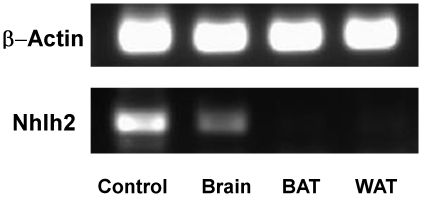
Nhlh2 expression WAT, Hypothalamus and BAT. Ethidium bromide-stained agarose gel showing PCR results following a 40-cycle amplification of mouse genomic DNA (control) or RNA from hypothalamus (Brain), white adipose (WAT), and brown adipose (BAT). β-actin expression was used as a loading control.

PCR of cDNA, using a high cycle number was carried out to determine if Nhlh2 is expressed in mRNA from either BAT or WAT. As shown in Figure 4, Nhlh2 is strongly expressed in mRNA from hypothalamus, as previously described [24], [34]. However, after 40 cycles of qRT-PCR, Nhlh2 is undetectable in mRNA from BAT and WAT.

### WAT Pro-inflammatory gene expression profile

**Figure 5:**
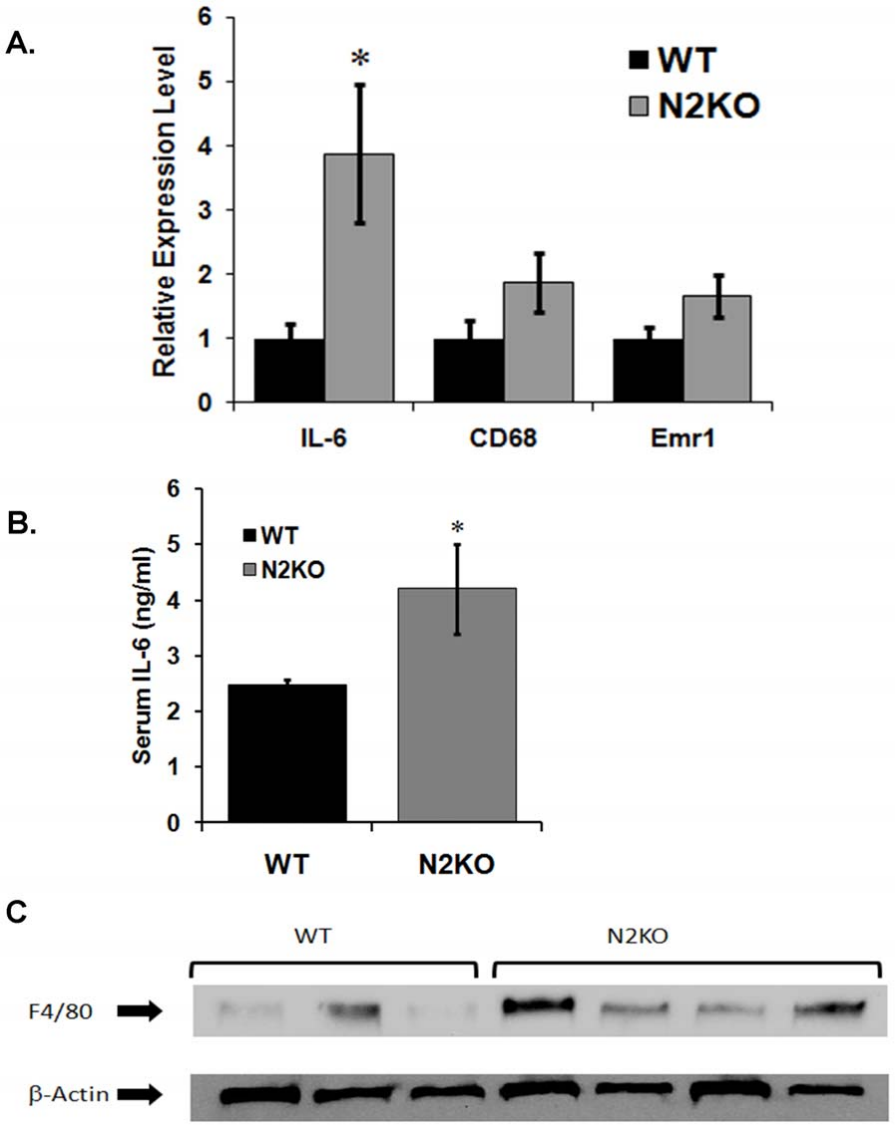
Expression profile for pro-inflammatory genes from WT and N2KO mice. **A**. Relative quantitative expression in RNA from WAT isolated from ad lib fed WT and N2KO for interleukin-6 (IL-6), Cluster of Differentiation 68 (CD68) and EGF-like module-containing mucin-like hormone receptor-like 1 (Emr1). The data are reported as mean expression level relative to WT expression ± SEM. (*p≤0.05). **B**. Serum IL-6 levels were measured from ad lib fed WT and N2KO mice. The data is reported as the serum level (pg/ml) ± SEM (*p≤0.05). **C.** Western analysis of WAT for F4/80 expression in protein extracted from ad lib fed mice. Equal total protein amounts were added for N = 3 WT mice, and N = 4 N2KO mice.

Considering the altered morphology of WAT from N2KO mice, combined with the fact that Nhlh2 is not expressed in WAT, the possibility of indirect mechanisms were explored, including macrophage infiltration. RNA isolated from WT and N2KO WAT was used to measure specific inflammatory and macrophage markers. Interleukin-6 (IL-6) mRNA expression was 3.87-fold higher in N2KO mice (Fig 5A, *p≤0.05, T-value = 2.31,df  = 7), indicative of an inflammatory state. WAT from N2KO mice showed a trend for higher expression levels of the macrophage markers EGF-like module-containing mucin-like hormone receptor-like 1 *(Emr1)* (1.66-fold higher) and Cluster of Differentiation 68 (*CD68*) (1.87-fold higher) in N2KO mice respectively when compared to WAT from WT mice (Fig. 5A). Consistent with increased IL-6 mRNA expression, serum IL-6 levels were also significantly increased (*p≤0.05, T-value = −2.12, df = 12, Fig 5B).

### F4/80, macrophage specific marker protein expression in WAT

To further examine the possibility of immune cell infiltration to WAT in N2KO mice, protein expression of macrophage marker F4/80 was measured. As predicted based on the increased levels of inflammatory proteins expressed in WAT, this macrophage specific marker is easily detectable in all samples from N2KO WAT, as compared to WAT from WT mice which showed only minimal expression of the F4/80 marker (Fig 5C). Immunohistological staining performed using the macrophage specific marker F4/80 shows increased protein in WAT of N2KO mice (mean densitometry reading of 48.6±35.4 units, WT versus 119.7±32.7 units, N2KO) (Fig 5C). While these increases do not reach the level of significance (p = 0.07, T-value  = 2.01, df = 5), they are supportive of the histological finding of macrophage infiltration into WAT.

### Adrenergic receptors and uncoupling protein expression in adipose tissue

Activation of the sympathetic nervous system is required to enter torpor as well as to initiate lipid mobilization [35], [36]. Expression of β-ARs are required for a normal torpor response [37] while β-ARs and uncoupling protein (UCP) family members expression in adipose tissue are necessary for thermogenesis responses [22], [23]. The expression of β-AR family members and UCPs were measured to determine if SNS input to BAT and WAT was affected in N2KO mice.

**Figure 6:**
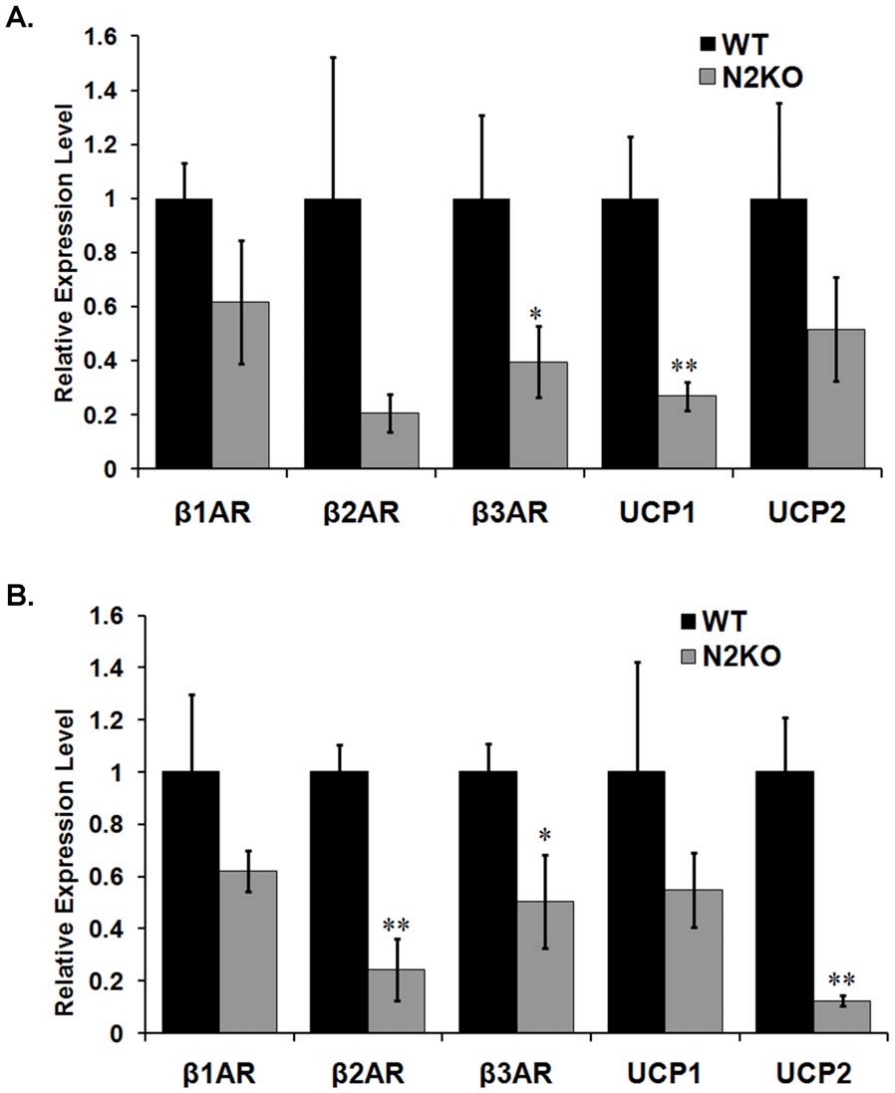
Expression profile in BAT and WAT. Relative quantitative expression levels for b-adrenergic receptors (β1AR, β2AR and β3AR) and uncoupling proteins (UCP1 and UCP2) in RNA isolated from BAT (**A.**) and WAT (**B.**). The data is reported as the mean expression level relative to WT expression ± SEM. (*p≤0.05, **p≤0.01).

In BAT β3-AR (*p≤0.05, T-value = 1.99, df = 6) were significantly down-regulated in N2KO mice compared with WT mice (Fig 6A). Levels of β2-AR trended towards being lower in N2KO mice but the high variability of β2-AR expression in WT mice prevented significant differences. Additionally, UCP-1 mRNA levels were significantly reduced by 4-fold in N2KO mice (**p<0.01, T-value = 3.31, df = 6). N2KO mice showed a reduced trend in mRNA expression levels of β1-AR, β2-AR and UCP-2 compared to WT mice (Fig 6A).

In WAT, β2-AR (**p≤0.01, T-value = -5.25, df = 10) and β3-AR (*p≤0.05, T-value = −4.46, df = 10) mRNA levels were significantly down-regulated by 4-fold and 2-fold respectively in N2KO mice compared with WT mice (Fig 6B). Also, UCP-2 mRNA levels were reduced by 5.63-fold in WAT from N2KO mice compared to WT mice (**p≤0.01, T-value = 4.13, df = 4). Both β1-AR and UCP-2 mRNA trended toward reduced levels in N2KO mice compared to WT mice levels (Fig 6B).

### Metabolic gene profile in WAT

**Figure 7:**
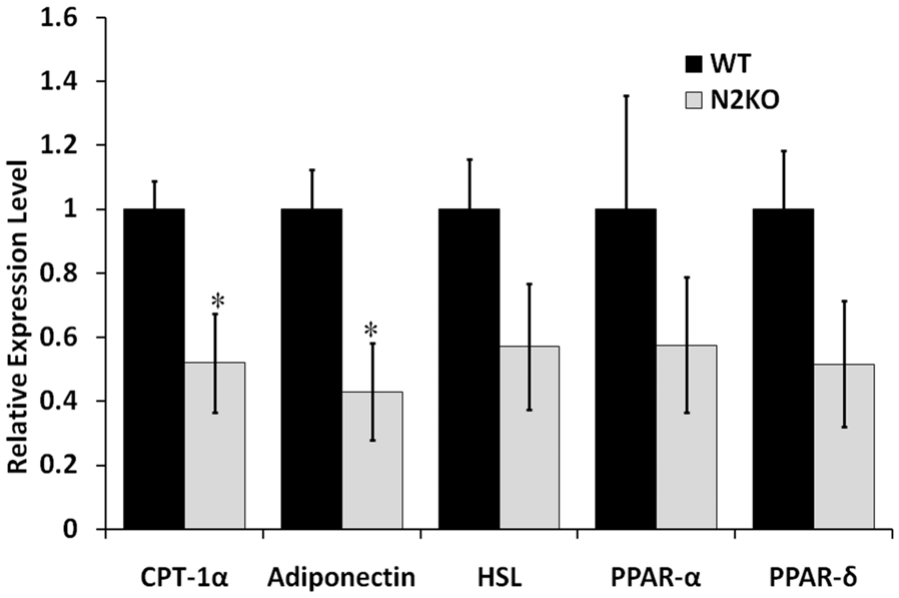
WAT metabolic gene expression profile in WT and N2KO mice. Relative quantitative expression levels of carnitine palmitoyltransferase-1α (CPT-1α), adiponectin, hormone sensitive lipase (HSL), and peroxisome proliferator-activated receptor alpha (PPARα) and delta (PPARδ) in WAT from ad lib fed WT and N2KO. All samples were normalized to β-actin expression. The data is reported as the mean expression level relative to WT expression ± SEM. (*p≤0.05, **p≤0.01).

In addition to entrance into torpor and lipid mobilization, the SNS controls important functions such as lipolysis in WAT [35], [38]. The mRNA level of genes involved in lipid metabolism, such as carnitine palmitoyltransferase-1α (CPT-1α), hormone sensitive lipase (HSL), peroxisome proliferator-activated receptor-α, and δ (PPAR-α, PPAR-δ) and adiponectin, were measured. CPT-1α (*p≤0.05, T-value = 2.91, df = 7) and adiponectin (*p≤0.05, T-value = -3.23, df = 7) were down regulated in N2KO mice compared with matched WT animals. HSL, PPAR-α and PPAR-δ showed trends towards reduced expression in N2KO mice (Fig. 7).

## Discussion

There are many mouse models available to study the mechanisms of obesity [39]. However, only a few of these models can help elucidate the transcriptional mechanisms controlling the CNS regulation of fat metabolism. A considerable amount of work confirming the role of the SNS in neural control of adipose tissue has been conducted [6], [18], [19], [40], [41]. Data presented herein shows that whole-body deletion of Nhlh2 leads to phenotypic alternations in both brown and white adipose tissue, even though Nhlh2 is not expressed in either of these tissues. We provide evidence suggesting that the hypothalamic transcription factor Nhlh2 is necessary for SNS-mediated control of BAT and WAT. Lack of Nhlh2 leads to generalized inflammation and a defective torpor response in the mutant animals.

We have repeatedly reported normal serum leptin levels in 8-week old pre-obese N2KO mice [29], [32]. In order to assess conditions resulting in differential leptin levels in our pre-obese mutant animals, leptin levels in food-deprived N2KO and WT mice were determined. The normal physiological response to fasting in animals is a lowering of circulating leptin levels. However, leptin levels are elevated in fasted N2KO mice. Conditions such as fasting or cold exposure normally stimulate WAT's sympathetic drive and decreases leptin synthesis/release in mice [10], [11], reducing leptin expression in isolated adipocytes [42]. These processes are necessary conditions for torpor to occur [31]. We have previously reported that N2KO mice also do not reduce circulating leptin levels following 24-hour exposure to cold [29]. Taken together, these data suggest that N2KO mice have a defective torpor response.

To further explore the defective torpor phenotype in N2KO mice, we examined the expression of β-ARs. β-ARs are expressed predominantly in adipose tissues [43], [44], [45], [46], [47], and are required for fasting-induced torpor in mice, as β3-AR antagonists block a normal torpor response [31], [37]. Here, we report that N2KO mice express β2-AR and β3-AR mRNA at lower levels in both BAT and WAT indicating lowered SNS input from the CNS. This is consistent with earlier findings that N2KO mice have severely reduced innervation of WAT including sensory and autonomic nerves [36]. Although β3-AR knockout mice develop obesity at an early age (3 weeks), this is without hyperphagia, which is similar to N2KO mice and suggests an impairment of energy expenditure, rather than energy intake pathways [27], [48], [49].

The aberrant adipose phenotype of N2KO mice includes morphological tissue changes. BAT from N2KO mice appears packed with larger lipid vacuoles compared with the dense, small lipid vacuoles in BAT from WT mice. This appearance is similar to that shown by Bachman and colleagues for β—less AR triple knockout mice, although the authors of this paper state that the BAT from β1-β3 double knockout mice appears normal [50]. Furthermore, in N2KO mice, WAT histology indicates the presence of smaller dense cells infiltrating between the adipose cells. Evidence of slightly higher levels of macrophage-specific markers, as well as significantly increased levels of serum IL-6 and mRNA, suggest an inflammatory state for N2KO WAT. Our results are consistent with the reduced peripheral innervation and vascularization of adipose tissue in N2KO mice previously shown to lead to preadipocyte and macrophage infiltration to WAT and changes in tissue architecture [36].

SNS controls many adipose tissue functions including lipolysis and lipogenesis, and reduced innervation leads to reduced lipolysis and increased lipogensis [6], [19], [35]. In the present study, reduced mRNA expression of CPT1-α and HSL is indicative of reduced lipolysis. Likewise, the reduction of adiponectin, PPAR-α and PPAR-δ gene expression contributes to our contention that the adipose tissue from N2KO mice has abnormal functionality. Indeed, the inflammatory state of WAT in N2KO mice is consistent with earlier findings that adiponectin is inversely related to increased IL-6 levels [51].

In summary, these results support a role for Nhlh2 in adipose tissue expression of bAR expression, perhaps through the sympathetic nervous system. Reduced adrenergic tone in adipose tissue leads to inflammation, altered morphology of both brown and white adipose tissue, and aberrant expression of uncoupling proteins in these tissues. These results provide an explanation for the abnormal leptin response and failure of torpor induction following both fasting and cold exposure. Nhlh2 is not expressed in WAT or BAT suggesting that the transcriptional activity by Nhhl2 on specific genes within the CNS is affecting the peripheral adipose-specific metabolic response. One of these Nhlh2-target genes is the neuropeptide processing enzyme PC1/3 [26]. Reduced PC1/3 levels in N2KO mice results in reduced expression of melanocortin levels throughout the hypothalamus. As the melanocortin signaling pathway has been implicated in sympathetic nervous system activity through β-ARs and WAT metabolism, our studies suggest a link between transcriptional regulation of melanocortin pathway genes by Nhlh2 and downstream peripheral effects leading to abnormal adipose functioning.

## Materials and Methods

### Animals

All animal protocols were approved by the Institutional Animal Care and Use Committee at Virginia Polytechnic Institute and State University, or the University of Massachusetts-Amherst (leptin measurements, body temperature and adipose histology). Animal colony maintenance, breeding and genotyping have been previously described [24]. N2KO and WT mice were maintained in 12 hr light, 12 hr dark conditions with *ad libitum* (ad lib) access to food (4.5% crude fat). At 14 weeks all mice were euthanized by CO_2_ asphyxiation at 1300 hr to standardize hormone and steroid levels that fluctuate hourly. For the fasting studies male WT and N2KO mice were individually housed in hanging wire-bottom cages and given free access to food and water for 48 hours prior to the start of the experiment. All experiments began at 11 AM on the day of testing (day 1). Animals with *ad lib* food received a measured amount of approximately 30 grams of food. All other animals began a 24 hour fast until 11 AM the following day. At 11 AM on day 2, body temperature was measured using a Thermalert TH-5 mouse rectal probe attached to a Physitemp (Clifton, NJ) digital thermometer. All mice are euthanized by 1 PM. Blood was collected by exsanguination and serum was used for leptin and Il-6 assays (below).

### Histology

WT and N2KO mice were euthanized under *ad libitum* conditions. Brown adipose tissue (BAT) was isolated by dissecting the interscapular brown fat depot. White adipose tissue was dissected by removing the visceral intra-abdominal fat pad from mice. Tissue was fixed overnight in the tissue fixative Histochoice (Amresco, Solon, OH) and then embedded in paraffin blocks at the Pioneer Valley Life Sciences Institute (Springfield, MA) or at the AML Laboratories Inc. (Rosedale MD). 6 µm sections were placed on glass slides (VWR Superfrost Plus, West Chester, PA). After staining with Hematoxylin and Eosin, slides were cover-slipped. Slides were examined under an Olympus BH-2 microscope (Olympus, Melville, NY) with the same exposure time, brightness and contrast for comparison groups. 40X and 10X images were taken. For both BAT and WAT adipose tissue, N = 3 WT and N = 3 N2KO mice examined.

### qRT-PCR from white and brown adipose tissue to detect gene expression

Intra-abdominal visceral WAT was collected from WT (N = 7) and N2KO (N = 11) 14-weeks old mice. Intrascapular BAT was collected from WT (N = 6) and N2KO (N = 7)) 14-weeks old mice. Tissues were homogenized into 4 M guanidine isothiocyanate buffer. Samples were layered over 5.7 M cesium chloride buffers and spun for 18 h at 120,000×*g* at 20°C. The supernatant was discarded, and RNA was resuspended in water and stored frozen until use. RNA was then DNAse treated prior to cDNA preparation. cDNA was created using reverse transcriptase in a magnesium buffer (Promega Corp.) for 1 hr at 42°C. qRT-PCR was performed using *Power* SYBR® Green, PCR master mix (2X). mRNA levels of each gene of interest was normalized against β-actin. A list of primer sequences used for amplification is found in Table 1. β-actin levels are constant between WT and N2KO animals in all energy states [34]. Normalized levels of mRNA were measured in triplicate per individual mouse from which sample means were calculated for each mouse. Data is presented as the fold-difference relative to the WT control group. For each mRNA amplified, melting-curve analysis was done to confirm the presence of a single amplicon.

**Table 1:** Primer sequences used for quantitative real-time RT-PCR assays.

Gene	Direction	Oligonucleotide sequence 5′>3′	Accession No.
Adiponectin	Forward	TGTTCCTCTTAATCCTGCCCA	NM_009605
	Reverse	CCAACCTGCACAAGTTCCCTT	
β-actin	Forward	GGAATCCTGTGGCATCCAT	NM_007393
	Reverse	GGAGGAGCAATGATCTTGATCT	
β1-AR	Forward	GCTGCAGACGCTCACCA	NM_007419
	Reverse	GCGAGGTAGCGGTCCAG	
β2-AR	Forward	CACAGCCATTGCCAAGTTCG	NM_007420
	Reverse	CGGGCCTTATTCTTGGTCAGC	
β3-AR	Forward	AGACAGCCTCAAATGCATCC	NM_013462
	Reverse	CCCAGTCCACACACCTTTCT	
CD68	Forward	CACCACCAGTCATGGGAATG	NM_009853
	Reverse	AAGCCCCACTTTAGCTTTACC	
CPT-1α	Forward	AAAGATCAATCGGACCCTAGACA	NM_013495.1
	Reverse	CAGCGAGTAGCGCATAGTCA	
Emr1	Forward	TTGTACGTGCAACTCAGGACT	NM_010130
	Reverse	GATCCCAGAGTGTTGATGCAA	
HSL	Forward	-CCT CAT GGC TCA ACT CC	NM_010719.5
	Reverse	GGT TCT TGA CTA TGG GTG A	
Nhlh2	Forward	CAG TTG GCG TGA AGA GGT AGA	NM_178777.2
	Reverse	AATGCCCACGAGAAATACCA	
PPAR- α	Forward	TGGGGATGAAGAGGGCTGAG	NM_011144.3
	Reverse	GGGGACTGCCGTTGTCTG	
PPAR-δ	Forward	ACAGTGACCTGGCGCTCTTC	NM_011145.3
	Reverse	TGGTGTCCTGGATGGCTTCT	
UCP-1	Forward	AAACAGAAGGATTGCCGAAA	NM_009463
	Reverse	TGCATTCTGACCTTCACGAC	
UCP-2	Forward	CTACAAGACCATTGCACGAGAGG	NM_011671
	Reverse	AGCTGCTCATAGGTGACAAACAT	

All sequences are to the mouse genes, and the forward and reverse primers are indicated.

### Western blot of white adipose tissue to detect F4/80 protein expression

For F4/80 protein analysis, abdominal adipose tissue was collected (N = 3 WT and N = 4 N2KO) in RIPA buffer, homogenized and processed for Western analysis using the published methods [52]. Equal amounts of protein (20 µg/lane), as determined using BCA Protein Assay (Pierce, Thermo Scientific, Rockford, IL) was run on a 8% SDS polyacrylamide gel and transferred to nitrocellulose membrane. Western blotting was performed using a rat monoclonal F4/80 antibody (Abcam, Cambridge, MA) with rabbit anti-rat HRP linked antibody as a secondary antibody. Chemoluminescent signal (ECL, Pierce, Rockford, IL) was detected.

### Serum protein measurements

Serum leptin was measured using the mouse leptin ELISA (Quantikine M Mouse Leptin immunoassay, R&D 30 Systems, Minneapolis, MN) on trunk blood collected from *ad libitum* fed (N = 6, each genotype) or 24-hour fasted (N = 5, each genotype) mice. Serum IL-6 levels were measured by ELISA (Mouse IL-6 Ready-SET-Go! ELISA Kit, eBiosciences, Inc. San Diego, CA) in serum separated from the blood collected from *ad libitum* fed mice WT (N = 7) and N2KO (N = 11).

### Non-quantitative Polymerase Chain Reaction

RNA was isolated from WAT, hypothalamus and BAT by guanidine isothiocyanate preparation and resuspended at 100 ng/µl. RNA (1μg) was DNase-treated prior to cDNA preparation. cDNA was created using reverse transcriptase in a magnesium buffer (Promega Corp.) for 1 hr at 42°C. PCR was performed on 2μl of DNAse-treated cDNA, or mouse genomic DNA (control) in a 25 ml reaction volume using Taq DNA Polymerase (Qiagen, Valencia, CA) and gene specific oligonucleotide primers (TABLE 1). Forty cycles of 1 min at 94°C, 1 min at 57°C and 1 min at 72°C with the extension time of 10 min at 72°C was performed to amplify.

### Statistical analysis

All values are expressed as mean ± SEM unless indicated otherwise. Comparison of means between two groups was made using unpaired two-tailed Student's T-test. *P* and *T* values were calculated using statistical analysis function in Microsoft Excel® (2007 version). Significance is expressed at *p≤0.05; **p≤0.01.
